# The Association of General and Central Obesity with Dietary Patterns and Socioeconomic Status in Adult Women in Botswana

**DOI:** 10.1155/2020/4959272

**Published:** 2020-09-01

**Authors:** Boitumelo Motswagole, Jose Jackson, Rosemary Kobue-Lekalake, Segametsi Maruapula, Tiyapo Mongwaketse, Lemogang Kwape, Tinku Thomas, Sumathi Swaminathan, Anura V. Kurpad, Maria Jackson

**Affiliations:** ^1^National Food Technology Research Centre, Department of Nutrition and Dietetics, Private Bag 8, Kanye, Botswana; ^2^Michigan State University, Alliance for African Partnership, 427 N Shaw Lane, East Lansing, Michigan 48824, USA; ^3^Botswana University of Agriculture and Natural Resources, Department of Food Science and Technology, Private Bag, 0027 Gaborone, Botswana; ^4^University of Botswana, Department of Family and Consumer Sciences, Private Bag, 0022 Gaborone, Botswana; ^5^Ministry of Health & Wellness, Private Bag, 0038 Gaborone, Botswana; ^6^St Johns Research Institute, Koramangala, Bengaluru, Karnataka, India; ^7^University of the West Indies, Department of Community Health and Psychiatry, Kingston, Jamaica

## Abstract

Dietary patterns and their association with general and central obesity among adult women were studied using a cross-sectional survey with multistage cluster sampling in urban and rural areas nationwide in Botswana. The participants in the study were adult women (*N* = 1019), 18–75 years old. The dietary patterns were identified using principal component analysis, and their associations with the body mass index and the weight-for-height ratio were examined. Factor analysis with varimax rotation was used to identify six dietary patterns (fast foods, refined carbohydrates, vegetables and fruits, fish and nuts, Botswana traditional foods, and organ and red meat dietary pattern). Overall, 24.5% of the women were overweight (BMI 25.0–29.99 kg/m^2^) and 24.5% were obese (BMI > 30 kg/m^2^). A waist-to-height ratio greater than 0.5 was observed for 42.2% of the women. With adjustment for age and education, individuals in the highest tertile of the Botswana traditional food pattern had a significantly higher risk of general obesity (RR = 1.40, 95% CI: 1.07–1.84) and central obesity (RR = 1.20, 95% CI: 0.97–1.48). With respect to the fish and nut pattern, a significant association was observed with central obesity only (RR = 1.43, 95% CI: 1.18–1.72). The Botswana traditional food pattern, characterised by a high carbohydrate intake, was found to be associated with a high risk of obesity in this study. However, more research is required to assess other factors contributing to obesity in women so that appropriate intervention programs can be put in place to help control this epidemic.

## 1. Introduction

The rise in the prevalence of obesity throughout the world is reflecting the strong impact of lifestyle factors including diet in its aetiology [1]. Although diet has been identified as the most predominant factor leading to imbalance between caloric intake and output, several other factors such as psychological, hereditary, and environmental factors have been linked to overweight and obesity [2]. Dietary patterns in African populations seem to be changing with urbanization and have presumably been characterised by adoption of Western lifestyles [3]. Obesity is associated with a wide range of pathological conditions such as hypertension, type 2 diabetes, cardiovascular disease, and early death [4–6]. High prevalence rates of obesity have been reported in Botswana women [7, 8]. These high rates call for accelerating programs that target primary prevention, a practice that requires local data on the magnitude of the problem and associated risk factors. It is therefore of particular interest to identify subpopulations at a high risk of overweight or obesity and identify risk factors contributing to this in order to generate information that could be useful in guiding the implementation of programs for reducing obesity in the affected population groups.

Numerous studies have examined relationships between single nutrients, particularly dietary fat, and obesity, but dietary determinants of weight gain remain controversial [9]. However, since foods are consumed in combinations, several authors have proposed analysing food consumption as dietary patterns [10–12] because they describe the whole diet, including the potential synergic effects of foods or nutrients [13]. A factor analysis approach has been most widely used to derive dietary patterns. This approach decreases the complexity of diets to a few important foods and has the ability to integrate complex and subtle interactive effects of many dietary exposures [14]. Moreover, by using this method, it is possible to examine distinct dietary patterns that may be related to the development of obesity. Exploring the association between dietary patterns and obesity will assist in tailoring public health programs aimed at combating this epidemic. The aim of this paper is to identify dietary patterns and socioeconomic status and explore if these are related to overweight and obesity. This is the first study which reports anthropometric and socioeconomic variables and dietary intake associated with excess adiposity in women in a Southern African country, Botswana.

## 2. Methods

### 2.1. Sample and Study Design

This was a cross-sectional study using a multistage cluster sampling design. Botswana is divided into 15 administrative districts, and from these, only nine (9) were selected to reflect a population distribution representative of urban and rural Botswana in its demographic and socioeconomic characteristics [15] (Central Statistics Office census data, 2001). A sample of towns, cities, and urban and rural villages was then randomly selected. Census enumeration areas (EAs) were used for household selection, and the number selected was proportional to the size of the enumeration area. At the final stage of sampling, every 40th household was selected until the required numbers of participants were recruited. The sample size required was 1500 adults who were aged 18 years and above, but data were collected from 1406 adults based on the agreement of the head of each household and the project resources. Data were collected by interviewers who were trained by nutrition researchers at the National Food Technology Research Centre (NFTRC) and the University of Botswana (UB) prior to the study.

Participants gave written informed consent to participate in the study, and they were informed of the purpose of the study, the confidentiality of the information that will be provided, and their right to refuse or terminate participation at any time during the interview. The same criterion was applied in previous studies of African populations [16, 17]. Although data were collected from both men and women, there was a very small representation of men in the sample (*n* = 286 men vs. *n* = 1117 women), and on examination of data, it appeared that the diets of men were different from that of women. It was also observed that the prevalence of obesity was much higher among women than men. Hence, data on women alone were considered for this analysis.

### 2.2. Ethical Considerations

The study was approved by the Ethics Committee of the Ministry of Health and Wellness in Botswana, and subjects gave written informed consent to participate in the study.

### 2.3. Data Collection Procedures

#### 2.3.1. Sociodemographic Variables

Participants gave written informed consent to participate in the study, and they were informed about the purpose of the study, the confidentiality of the information that will be provided, and their right to refuse or terminate participation at any time during the interview. All questionnaires were interviewer administered and were adapted from the National Food Consumption Survey conducted in South Africa [18]. The variables collected for the socioeconomic questionnaire included place of residence, age, gender, education level, and number of possessions. In the questionnaire, educational status was evaluated by asking the participants to declare their highest level of educational achievement. Education level was grouped into three categories: (a) illiterate and primary education, (b) secondary education, and (c) tertiary education. Place of residence was categorised into three groups, namely, towns, urban villages (classified by the presence of Tribal Administration, District Council, and Central Government District Administration offices), and the other rural villages. The number of possessions was regarded as low if the household had 0-1 items, medium for 2-3 items, and high for 4-5 items.

#### 2.3.2. Anthropometric Measurements

Anthropometric measurements were taken using standardized techniques and calibrated equipment. Body weight was recorded to the nearest 0.1 kg on a digital scale (SECA model 803, SECA Corp., Hamburg, Germany, 2000). Standing height was measured without shoes on a floor standing stadiometer (SECA model 213, SECA Corp Hamburg, Germany, 2000) to the nearest 0.1 centimetres. Body mass index (BMI) was calculated as weight in kilograms divided by the square of height in metres. The World Health Organization (WHO) classification of BMI was used to determine overweight and obesity [19]. Waist circumference (WC) was measured at the narrowest level [20] using a nonstretchable tape measure, without any pressure to body surface; measurements were recorded to the nearest 0.1 cm. Central obesity was defined as WC ≥ 88 cm [19]. Waist-to-height ratio (WHtR) was calculated as waist (cm) divided by height (cm). Raised waist-to-height ratio (WHtR) was defined as > 0.5 [21] and is used as a cutoff point for abdominal obesity risk. All measures were taken in duplicate, and the average was used in the analysis.

#### 2.3.3. Dietary Intake Assessment

A validated quantitative food frequency questionnaire (QFFQ) as described in an earlier manuscript [22] was administered for assessing usual dietary intakes. The questionnaire included 122 food and drink items. The FoodFinder, a South African food composition database, was used to calculate the nutrient composition of the habitual diet [23]. The QFFQ was calibrated against four nonconsecutive 24-h recalls administered over 12 months in a subsample of the total population. Spearman rank correlation coefficients for energy-adjusted nutrients ranged from 0.42 (carbohydrate) to 0.49 (protein) for macronutrients and 0.23 (iron) to 0.44 (polyunsaturated fat) for micronutrients when compared with the 24-h recalls. Exact agreement for quartile distribution between the QFFQ and recalls for nutrients ranged from 27% to 72%. Weighted kappa values were the lowest for retinol (0.13), iron (0.22), and beta-carotene (0.25) and ranged from 0.33 (saturated fat) to 0.59 (folate) for other nutrients (energy, carbohydrate, protein, fat, calcium, vitamin E, and fibre), thus showing reasonable comparison of these methods. The reproducibility of the QFFQ was assessed, and Pearson's correlation coefficients (energy adjusted) varied between 0.39 for retinol and 0.66 for vitamin E with most values falling between 0.50 and 0.60 from the first and second measurements (22).

Frequency of usual food consumption was estimated using one of the 8 precoded categories of responses. For each food item, participants were asked to supply information on portion size by using food models, commonly used household utensils, measuring cups, and a measuring tape to indicate the portion size usually consumed. Daily nutrient intakes were calculated from the questionnaire by multiplying the frequency of use by the nutrient composition specified for each food item and its portion weight. The values obtained were summed up to obtain a whole day's intake. The daily energy and nutrient consumption by participants was computed using the South African Food Composition database [22]. To reduce measurement errors, all nutrient intakes were adjusted for total energy by regressing nutrient intakes on total energy intake, and these values were used for analysis using a computer programme written for SPSS. The coefficients of 0.0, 0.03, 0.08, 0.14, 0.40, 0.80, 1.00, and 2.5 were used to indicate frequencies of almost never, once per month, 2-3 times per month, once per week, 2–4 times per week, 5-6 times per week, once per day, and 2 or more times per day, respectively. Nutrients from all foods were summed to obtain a total nutrient intake for each individual.

For the purposes of determining of dietary patterns, 122 food items were grouped into 23 food groups (Table 1) based on the similarities in ingredients and the particular food item nutrient profile. Food items having a unique composition (e.g., eggs) were presented individually. Daily intake in grams of the foods in each group was used for analysis.

### 2.4. Statistical Analyses

Data were double entered and cleaned. From the data obtained, participants who reported daily energy intake less than 800 kcal and more than 5500 kcal were excluded. The same criterion was applied in previous studies of African populations [16, 17]. Descriptive statistics was applied, and proportions and percentages are reported. Principal component factor analysis (PCFA) was used to identify dietary patterns using intakes of the twenty-three food groups. The factorability of the correlation matrix was supported by the significance of Bartlett's test of sphericity [24] and the scree plot. Factors that had at least 3 items and with eigenvalues more than 1 were considered. The scree plot was examined to identify a breakpoint (where the plot starts flattening) in the eigenvalues, and the breakpoint was observed to appear after six factors. In order to confirm the choice of 6 as the best number of factors, factor analysis was repeated twice by manually fixing 5 and 7 factors (one number below and above 6). Examination of these analysis confirmed that 6 was the best number of factors. The factors were orthogonally transformed by using varimax rotation to achieve independent (nonoverlapping) factors and with greater interpretability. To interpret the results and provide a label for a given pattern, we considered the items most strongly related to that pattern, i.e., those for which the absolute value of the loading coefficient (which is the correlation of each variable with the given dietary pattern) was >0.30 [24]. The factor scores for each subject on each of the patterns were computed and were categorised as 0 and the remaining as tertiles 1, 2, and 3. The value 0 represented the lowest intake of a dietary pattern (factor) for a subject, and 3 represented the highest intake of a dietary pattern with 1 and two in between. A chi-square test was used to examine the association of dietary patterns with the different socioeconomic characteristics. In order to examine the effect of the different dietary patterns on overweight/obesity and central obesity, risk separate log-binomial regression analyses were performed. The probable confounders for these associations such as age, education level, place of residence, and number of possessions were identified using the chi-square test. All parameters that were significant at 10% level were considered for the multivariate model. Separate multiple log-binomial regression analyses were employed to identify the risk of central obesity risk with respect to the dietary patterns while adjusting for significant confounders. Total energy intake of the subjects was also included in the analyses. Risk ratio (RR) with corresponding 95% confidence interval (95% CI) and *p* values were reported. The data were analyzed using SPSS version 20 (SPSS). Log-binomial regression analysis was carried out using the PROC GENMOD program in SAS software version 9.2 (SAS). *p* < 0.05 was considered statistically significant.

## 3. Results

### 3.1. Socioeconomic Characteristics and Dietary Patterns

The socioeconomic and anthropometric characteristics of the participants are presented in Table 2. A total of 1117 women participated in this dietary patterns study. The average age was 37.6 ± 16.4 years. The mean BMI was 26.0 ± 6.1 kg/m^2^, and 49.7% of women were overweight or obese. Specifically, 42% of the women had normal BMI, whilst 25.0% were overweight and 24.7% were obese. The mean waist circumference was 78.6 cm, and 22.7% were above the abdominal adiposity cutoff value of 88 cm. Just over 42% were above the cutoff value of abdominal adiposity risk according to the WHtR (0.5) and mostly lived in urban villages. Almost all of the women had junior secondary education and had at least 2-3 possessions.

**Figure 1:**
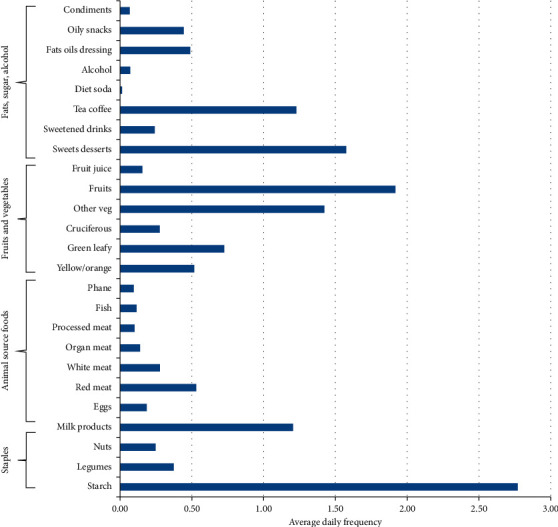
Frequency of consumption of the food groups.

Figure 1 shows the daily frequency of consumption of the different food groups. The most frequently consumed foods were starchy foods (almost three times daily), followed by fruits which were eaten almost twice daily. Sweets, desserts, and other vegetables (e.g., cucumber and mushroom) were eaten on average once a day. Milk and milk products and hot beverages such as tea and coffee were also consumed close to one and a half times daily. Foods that included diet soda, insects, fish, organ meats, and processed meats were reported to be less frequently consumed.

The factor loadings of food groups on the six dietary patterns are shown in Table 3. The six factors together accounted for 41.9% of the total variance. The patterns were named according to the food groups loading the highest on the respective dietary pattern. However, it should be noted that there is a possibility for the patterns to be labelled differently by other researchers. These were labelled as follows: (i) fast foods: principally characterised by a higher consumption of savouries and fried snacks, eggs, sweetened drinks, white meat, fats, and oils; (ii) refined carbohydrate pattern: characterised by greater intakes of hot beverages, sweets and desserts, and milk and milk products; (iii) vegetables and fruits characterised by high intakes of foods recognised as healthy including other vegetables, fruit and fruit juices, and yellow/orange vegetables; (iv) fish and nuts characterised by intakes of cruciferous vegetables, nuts, and fish; (v) Botswana traditional food pattern loaded with insects, green leafy vegetables, legumes, and starches; (vi) organ and red meat pattern loaded with organ meat and red meat.

### 3.2. Socioeconomic and Anthropometric Characteristics and Association with Obesity

Table 4 shows the association between socioeconomic and anthropometric characteristics with obesity. Age had a positive association with general and central obesity (*p*=0.037 and 0.003), respectively, up to 60 years of age. Conversely, education was inversely associated with both indices of adiposity. The proportion of obesity was the highest among participants with low and high levels of education and the lowest among the middle education level. The area of residence and the number of possessions owned by the participants in the study did not have any association with obesity and risk of central obesity.

In our sample, the mean BMI was 26.0 ± 6.1 kg/m^2^; 49.7% of women were overweight or obese, and central obesity was 42.2% (Table 1). There was no significant risk of obesity found when participants were categorised as normal, overweight, and obese or when overweight and obese individuals were combined. However, when obese participants were taken as one group and normal and overweight as another, participants with the highest intake of the Botswana traditional food pattern had a significantly higher risk of obesity as compared to those reporting the lowest intake (RR = 1.6, 95% CI: 1.21–2.10) (Table 5). This was possibly due to the high intake of refined starchy foods in this food pattern. The other dietary patterns did not show any significant risk of obesity.

The Botswana traditional food pattern continued to be significantly associated with obesity when adjusted for age (RR_Age adjusted_: 1.55, 95% CI: 1.18–2.04) as shown in Table 6. However, when the level of education was included into the model, the significance disappeared, which suggests that education is a significant confounder in this relationship. The effect of dietary patterns on the risk of abdominal obesity was also examined. A significant association was observed with high intakes of the fish and nut pattern and the Botswana traditional food pattern and obesity in univariate models. However, the association diminished in multivariate models.

## 4. Discussion

The objective of this study was to identify the major dietary patterns in Botswana and determine whether any of the dietary patterns was associated with general and central obesity in women. Our data shows that 24.7% of women included in this study were obese and that 42.2% have a waist-to-height ratio greater than 0.5. Similar prevalence rates of obesity have been observed before; for instance, the Botswana STEPS survey [7] revealed that 24.6% of women were obese, while Letamo reported that 23% of women were obese [8].

Six distinct dietary patterns were identified in this population, named as “fast foods” (savouries, fried snacks, eggs, sweetened drinks, white meat, fats, and oils), “refined carbohydrates” (hot beverages, sweets and desserts, and milk and milk products), “vegetables and fruits” (vegetables, fruit and fruit juices, and yellow/orange vegetables), “fish and nuts” (cruciferous vegetables, nuts, and fish), “Botswana traditional foods” (insects, green leafy vegetables, legumes, and starches), and “organ and red meat” (beef and organ meats). Of these, the Botswana traditional food dietary pattern was positively associated with BMI, while the vegetable and fruit pattern was positively associated with the waist-height ratio after adjusting for age. These associations disappeared after education was added to the model, showing that socioeconomic status (the surrogate for education) confounded the relationship as the proportion of obesity was the highest among women with low education. The relationship between education level and obesity has been observed in previous studies [8, 25–27]. These results suggest that women with medium level of education may be more concerned about obesity and have higher awareness about the consequences of obesity and are more likely to live healthier lifestyles and eat healthier diets. Tzotzas and colleagues argue that education level does not reflect the current financial situation of a subject, but in most cases reflects its social status [26].

High rates of overweight and obesity among low-income populations have raised questions about whether food and nutrition assistance programs and or the cost of foods contribute to the problem. This may be the case in this study because the food basket for various programs targeting low socioeconomic class citizens in Botswana contains mainly sorghum meal, maize meal, vegetable oil, beans, and skimmed milk, which may be contributing to body weight gain in these women because of an increased starchy food intake [28]. Similar results were also observed among women who were Food Stamps recipients in the US. Since the inception of the Food Stamp Act in the USA, provision of food was guaranteed for those in poverty. This has however been coupled with the dramatic increase in the prevalence of obesity, particularly among those with low incomes [27]. Although the programs were designed to reduce poor nutrition, they may ironically lead to weight gain. Though programme participation may have positive and significant effects on the consumption of some foods, other nutrient needs are not always met [28].

The vegetable and fruit dietary pattern was significantly associated with central obesity (*p*=0.008). This finding is contradictory to previous observations that revealed a protective effect of these foods (fruits, fruit juices, and vegetables) included in this pattern [29]. The idea that fruit juices are healthy needs to be put into perspective; although the sugar in them is natural, it can lead to health challenges, like inability to maintain a healthy weight if taken in large quantities. The intake of fruit juices could also be compounding the effect because when the sugar is concentrated as in juices, the levels can be surprisingly high and potentially unhealthy [30]. A study conducted to assess the relative effects of glucose and fructose sugars during sustained consumption in humans observed that dietary fructose specifically decreases insulin sensitivity and increases visceral adiposity in overweight/obese adults [31]. Authors argue that the observed intra-abdominal fat gain suggest that fructose consumption may specifically promote lipid deposition in visceral adipose tissue (VAT), whereas glucose consumption appears to favour subcutaneous adipose tissue (SAT) deposition. More research is needed to understand the biological effects of these two major simple sugars in the diet and obesity development in humans.

Addressing the issue of obesity in African populations can be very challenging. According to Puoane et al., African women with larger size appear to be culturally more acceptable, portraying a symbol of health, wealth, sexuality, dignity, attractiveness, increased functional capacity, and absence of the human immune virus (HIV) [32]. These perceptions are also prevalent in Botswana and therefore may present complexities in the prevention and management of overweight and obesity [7]. Cultural influences on health attributions and beliefs and practices are well recognised; therefore, further research is warranted to understand the cultural beliefs that influence attitudes of Botswana women towards weight so that any strategies that could be employed to tackle this epidemic should take these into account. The HIV/AIDS pandemic is also affecting the perception of body size. Many women are becoming obese to avoid the stigma, shame, and discrimination, which are unfortunately still associated with being infected with HIV or having AIDS [32]. It will take a great deal of education and effort to change the perceptions of the African population here and elsewhere that being thin does not automatically mean that you are ill and that having a normal body weight is a desirable goal.

A major strength of our study is that dietary intake included a large number of food items, and apart from records reflecting extremes in energy intakes (outside the range of 800 to 5000 kcal), there were no further attempts to exclude diets from the analyses. Because of the higher physical activity levels of adults in developing countries, the average daily energy requirements are lower for populations living in developed than developing countries [33]; the same criterion was applied in earlier reports of diet and disease relationships among persons of the African origin [16, 34]. In this study, we adjusted for confounding variables (age and education) that were significantly related to weight status in univariate regression models. Notwithstanding, the reporting of diet may be related to recall bias or memory lapses, poor awareness of quantities or types of foods eaten [35], or inaccurate portion size estimation [36]. Over or understating dietary intakes, whether systematic and nonrandom, can result in incorrect assessments of the relationships between dietary components and patterns. Under reporting of energy intake or specific dietary components in obesity may also result in a specific bias in studies investigating the relationship between the aetiology and consequences of obesity [37, 38].

Though the relationship of dietary patterns with general obesity (BMI) has been studied in other populations, little is known about the effect of abdominal obesity (waist circumference and waist-to-hip ratio) on women of African descent. Central obesity has been previously reported to have greater discriminatory power in predicting the risks of obesity when compared with BMI [21]. To the best of our knowledge, these findings are one of the first evaluations of the association between dietary patterns identified by factor analysis and indices of adiposity in Botswana. However, several limitations should be considered when interpreting the results of this study. Caution should be exercised in assuming a causal relationship as these findings are from a cross-sectional study and not longitudinal data and may not reflect long-term patterns. Identification of dietary patterns using factor analysis is likely to be affected by several subjective decisions, including grouping of food items, number of factors to extract, method of rotation, and even the labelling of factors [39].

## 5. Conclusion

In conclusion, our evaluation of the associations between major dietary patterns and measures of adiposity in Botswana women provided insight into how the combination of foods and socioeconomic factors may increase the risk of obesity. Our results suggest that education level may influence the development of obesity. Although African countries are at the low economic level, obesity prevalence remains high in spite of the continuing battle against undernutrition. This confirms the emergence of the double burden of malnutrition described elsewhere [3, 35]. More information about the dietary patterns and nutrient intakes of this population, how these dietary exposures are related to health outcomes, and the forces in the food environment that drive eating behaviour will help to understand and address obesity development better and more comprehensively. A starting point could be to make sure that quality health statistics are generated in order to design interventions that will be tailor-made for Botswana women.

## Figures and Tables

**Table 1:** Food grouping used in the dietary pattern analyses.

Group	Items
(1) Starches	White & brown bread, *phaphatha*, rice, pasta, sorghum meal, mealie meal, baked, boiled, or mashed potatoes, sweet potato, *tsabana*, samp, mealie rice, *dikgobe, madombi*, breakfast cereals, fat cakes, millet, *mageu*, *and kabu*
(2) Legumes	Baked beans, red peas, black eye beans, *tswana* beans, texturized soy, soy milk, Bambara nuts
(3) Nuts	Peanuts and peanut butter
(4) Milk & milk products	Whole, evaporated, skimmed, powdered, and condensed milk, cream cheese, *madila, mayere*, cheese, ice cream, yoghurt, infant formula
(5) Eggs	Eggs
(6) White meat	Chicken with & without skin
(7) Red meat	Beef, lamb, *nama ya pudi,* minced meat, *seswaa*, beef biltong, pork
(8) Organ meats	*Serobe*, kidney, gizzard, liver
(9) Processed meats	Corned beef, sausages, pastarami, polony, bacon
(10) Fish	Fresh fish, canned tuna, canned fish, salted fish
(11) Yellow/orange vegetables	Butternut/pumpkin, carrots, *lerotse*
(12) Cruciferous vegetables	Cabbage, cauliflower, broccoli
(13) Green leafy vegetables	Spinach, lettuce, chomolia, rape, *morogo wa dinawa*
(14) Other vegetables	Cucumber, mixed veggies, beetroot, mushroom, *maraka*, peppers, *tswii*, avocado
(15) Fruits & fruit juices	Orange, naartjie, grapefruit, pawpaw, mango, banana, apple, peach, pear, watermelon, grapes, *morula*, dried fruit, canned fruit, kiwi, strawberry, pineapple, *moretlwa, mmilo*
(16) Sweets & desserts	Candies, sugar added to tea, cookies, cakes, pudding, jelly, custard, jam
(17) Sweetened drinks	Fruit drinks, sodas
(18) Diet sodas	Diet sodas
(19) Hot beverages	Tea, herbal tea, coffee, chocolate drink
(20) Alcoholic drinks	Wine, rum, whiskey, gin, beer, *chibuku*
(21) Fats, oils, & dressings	Butter, margarine, mayonnaise, salad dressing
(22) Savouries & fried snacks	Potato fries, cheese snacks, savoury meat pies
(23) Insect	*Phane*

**Table 2:** Characteristics of study participants.

	Females (*n* = 1117)
Area of residence: *n* (%)^*∗*^	
Town/city	363 (32.5)
Urban village	461 (41.3)
Rural village	293 (26.2)

Age: years (mean ± sd)	
Age categories *n* (%)	37.7 ± 16.3 (33.0)
18–24	291 (26.1)
25–34	292 (26.2)
35–44	212 (19.0)
45–59	184 (16.5)
≥60	137 (12.3)

Education^*∗*^	
No schooling	109 (9.8)
Primary	328 (29.4)
Secondary	565 (50.7)
Tertiary	112 (10.1)

Number of possessions^*∗*^	
0-1	408 (40.0)
2-3	447 (43.8)
4-5	166 (16.2)

Anthropometric measures (mean ± sd)	
Weight (kg)	66.29 ± 15.72
Height: (m)	1.60 ± 0.07
Body mass index (BMI) kg/m^2^:	26.0 ± 6.1

BMI categories	
<18.5 (underweight)	84 (7.6)
18.5–24.99 (normal)	471 (42.7)
25.00–29.99 (overweight)	276 (25.0)
≥30.00 (obese)	272 (24.7)

Central obesity	
Waist circumference (mean ± sd)	78.75 ± 14.12
WC, overweight 80–88 cm	222 (19.9)
WC, obese > 88 cm	252 (22.6)
Waist-height ratio (WHtR) > 0.5 (%)	465 (42.2)

^*∗*^Data presented as *n* (%) unless otherwise stated.

**Table 3:** Factor loading matrix for the six dietary patterns^a^.

	Fast foods	Refined carbohydrates	Vegetables & fruits	Fish & nuts	Botswana traditional foods	Organ & red meat
Savouries & fried snacks	0.617					
Eggs	0.598					
Sweetened drinks	0.554					
Fats, oil, & dressings	0.494					
White meat	0.431					
Hot beverages		0.695				
Sweets & desserts		0.686				
Milk & milk products		0.574				
Other vegetables			0.808			
Fruits & fruit juices			0.715			
Yellow/orange vegetables			0.592			
Fish				0.667		
Cruciferous vegetables				0.532		
Nuts				0.448		
Insect					0.554	
Green leafy vegetables					0.546	
Legumes					0.515	
Starches					0.451	
Organ meat						0.734
Red meat						0.712

^a^Food groups with factor loading values < 0.3 are excluded for simplicity.

**Table 4:** Social and demographic factors associated with the risk of general and central obesity.

	General obesity						Central obesity			
	Parameter	*N*	% obese	RR	95% CI	*p* value	% obese	RR	95% CI	*p* value
*Univariate model for obesity*										
Age (years)		1014	24.8			**0.037** ^*∗*^	41.8			**0.003** ^*∗*^
	≤25	303	22.4	Ref			37.7	Ref		
	26–45	436	22.7	1.02	0.78, 1.35	0.860	37.8	1.01	0.83, 1.22	0.951
	46–60	154	34.4	1.53	1.12, 2.07	0.007	54.8	1.46	1.19, 1.79	<0.001
	>60	121	25.6	1.15	0.80, 1.67	0.456	49.6	1.33	1.05, 1.67	0.016

Education level^†^		1014	24.8			**<0.001** ^*∗*^	41.8			**<0.001** ^*∗*^
	Low	393	35.9	2.31	1.82, 2.94	<0.001	60.7	2.12	1.81, 2.48	<0.001
	Medium	515	15.7	Ref			28.9			
	High	106	27.4	1.71	1.17, 2.49	0.005	34.9	1.20	0.89, 1.62	0.233

Area of residence		1014	24.8			**0.315**				**0.170**
	Town	330	26.7	Ref			39.4	Ref		
	Urban village	417	25.4	0.96	0.75, 1.23	0.753	45.6	1.15	0.97, 1.36	0.114
	Rural village	267	21.3	0.81	0.60, 1.08	0.151	39.0	0.99	0.81, 1.22	0.949

No of possessions		1011	24.7			**0.095**	41.8			**0.633**
	0-1	401	21.4	Ref			39.9	Ref		
	2-3	445	25.6	1.19	0.93, 1.52	0.167	42.9	1.07	0.91, 1.26	0.413
	4-5	165	30.3	1.39	1.03, 1.88	0.032	43.6	1.09	0.88, 1.22	0.431

^*∗*^Model significant at *p* ≤ 0.05 level. ^†^Education classified as low (no schooling and primary education); medium (secondary education); high (tertiary education).

**Table 5:** Univariate relative risks and confidence intervals for tertiles of dietary patterns associated with the risk of general and central obesity.

Parameter		*N*	% obese	RR	95% CI	*p* value	% at risk of central obesity	RR	95% CI	*p* value
Fast food pattern						**0.750**				**0.323**
	Level 1	601	25.8	Ref				**Ref**		
	Tertile 1	137	22.6	0.88	0.63, 1.23	0.459	0.8	0.89	0.70, 1.12	0.303
	Tertile 2	136	22.1	0.86	0.61, 1.21	0.383	0.9	0.94	0.75, 1.17	0.561
	Tertile 3	137	24.8	0.97	0.70, 1.33	0.830	0.7	0.82	0.67, 1.04	0.107

Refined carbohydrate pattern						**0.578**				**0.677**
	Level 1	633	23.5	Ref				**Ref**		
	Tertile 1	125	24.8	1.06	0.76–1.48	0.745	1.2	1.10	0.88, 1.37	0.407
	Tertile 2	127	29.1	1.24	0.92, 1.69	0.165	1.2	1.12	0.90, 1.39	0.304
	Tertile 3	126	26.2	1.12	0.81, 1.55	0.506	1.1	1.07	0.86, 1.34	0.558

Vegetable & fruit pattern						**0.878**				**0.004∗**
	Level 1	675	24.1	Ref				**Ref**		
	Tertile 1	112	24.1	1.00	0.70, 1.43	0.993	0.8	0.91	0.70, 1.18	0.471
	Tertile 2	112	26.8	1.11	0.80, 1.55	0.531	1.3	1.16	0.93, 1.45	0.202
	Tertile 3	112	26.8	1.11	0.80, 1.55	0.531	2.0	1.43	1.18, 1.72	<0.001

Fish & nut pattern						**0.233**				**0.294**
	Level 1	621	22.7	Ref				**Ref**		
	Tertile 1	129	30.2	1.34	0.99, 1.80	0.058	1.1	1.04	0.83, 1.30	0.744
	Tertile 2	132	25.8	1.14	0.82, 1.58	0.432	1.1	0.98	0.77, 1.23	0.844
	Tertile 3	129	27.9	1.24	0.90, 1.69	0.189	1.4	1.22	1.0, 1.49	0.051

Botswana traditional food pattern						**0.015∗**				**0.023∗**
	Level 1	603	22.1	Ref				**Ref**		
	Tertile 1	134	26.1	1.26	0.92, 1.72	0.152	1.4	**1.20**	0.97, 1.48	0.096
	Tertile 2	137	19.7	1.06	0.76, 1.49	0.722	1.2	1.13	0.91, 1.41	0.264
	Tertile 3	137	26.1	1.60	1.21, 2.10	<0.001	1.7	1.35	1.12, 1.64	0.002

Organ & red meat pattern						**0.503**				**0.594**
	Level1	585	25.3	Ref				**Ref**		
	Tertile 1	142	26.1	1.03	0.76, 1.41	0.836	0.8	0.88	0.70, 1.11	0.274
	Tertile 2	142	19.7	0.78	0.54, 1.12	0.181	0.9	0.95	0.76, 1.18	0.612
	Tertile 3	142	26.1	1.03	0.76, 1.41	0.836	0.8	0.89	0.71, 1.12	0.343

^*∗*^Model significant at *p* ≤ 0.05 level.

**Table 6:** Multivariate relative risks and confidence intervals for quartiles of food patterns associated with the risk of general and central obesity.

*Multivariate model for obesity adjusting for age*									

Botswana traditional food pattern					**0.023**				**0.059**
	Level 1		Ref	1			**Ref**		
	Tertile 1	134	1.25	0.92, 1.71	0.154	1.4	1.17	0.96, 1.44	0.129
	Tertile 2	137	1.07	0.76, 1.50	0.704	1.2	1.17	0.95, 1.45	0.148
	Tertile 3	137	1.55	1.18, 2.04	0.002	1.7	1.27	1.06, 1.53	0.014
Fish & nut pattern									**0.008**
	Level 1						Ref		
	Tertile 1	112				0.8	0.97	0.74, 1.26	0.805
	Tertile 2	112				1.3	1.15	0.93, 1.43	0.208
	Tertile 3	112				2.0	1.40	1.17, 1.68	<0.001

*Multivariate model for obesity adjusting for age, education, and total energy intake*									

Botswana traditional food pattern					**0.119**				**0.716**
	Level 1		Ref	1			**Ref**		
	Tertile 1	134	1.16	0.85, 1.57	0.347	1.4	1.15	0.85, 1.56	0.372
	Tertile 2	137	1.04	0.75, 1.44	0.827	1.2	1.03	0.74, 1.44	0.857
	Tertile 3	137	1.40	1.07, 1.84	0.013	1.7	1.42	1.08, 1.86	0.012
Fish & nut pattern									**0.236**
	Level 1						Ref		
	Tertile 1	112				0.8	1.04	0.75, 1.46	0.799
	Tertile 2	112				1.3	1.02	0.74, 1.41	0.882
	Tertile 3	112				2.0	0.89	0.65, 1.25	0.526

## Data Availability

The observational data used to support the findings of this study are available from the corresponding author upon request.
